# Sulky Dignity

**Published:** 1854-11

**Authors:** 


Sulky Dignity.—We sometimes meet with men who seem to think that any indulgence in an affectionate feeling is a weakness. They will return from a journey, and greet their families with a distant dignity, and move among their children with the cold and lofty splendor of an iceberg, surrounded by its broken fragments. There is hardly a more unnatural sight on earth than one of those families without a heart. A father had better extinguish a boy's eyes than take away his heart. Who that has experienced the joys of friendship, and values sympathy and affection, would not rather lose all that is beautiful in nature's scenery, than be robbed of the hidden treasure of his heart? Cherish, then, your heart's best affections. Indulge in the warm and gushing emotions of filial, parental and fraternal love. Think it not a weakness. God is love. Love God, everybody, and everything that is lovely. Teach your children to love; to love the rose, the robin; to love their parents; to love their God. Let it be the studied object of their domestic culture, to give them warm hearts, ardent affections. Bind your family together by these strong cords. You cannot make them too strong. Religion is love; love to God, love to man.—Anon.

